# Fire Severity Filters Regeneration Traits to Shape Community Assembly in Alaska’s Boreal Forest

**DOI:** 10.1371/journal.pone.0056033

**Published:** 2013-02-13

**Authors:** Teresa N. Hollingsworth, Jill F. Johnstone, Emily L. Bernhardt, F. Stuart Chapin

**Affiliations:** 1 USDA Forest Service, Pacific Northwest Research Station, Fairbanks, Alaska, United States of America; 2 Department of Biology, University of Saskatchewan, Saskatoon, SK, Canada; 3 Department of Biology and Wildlife, University of Alaska Fairbanks, Fairbanks, Alaska, United States of America; 4 Institute of Arctic Biology, University of Alaska Fairbanks, Fairbanks, Alaska, United States of America; USDA-ARS, United States of America

## Abstract

Disturbance can both initiate and shape patterns of secondary succession by affecting processes of community assembly. Thus, understanding assembly rules is a key element of predicting ecological responses to changing disturbance regimes. We measured the composition and trait characteristics of plant communities early after widespread wildfires in Alaska to assess how variations in disturbance characteristics influenced the relative success of different plant regeneration strategies. We compared patterns of post-fire community composition and abundance of regeneration traits across a range of fire severities within a single pre-fire forest type– black spruce forests of Interior Alaska. Patterns of community composition, as captured by multivariate ordination with nonmetric multidimensional scaling, were primarily related to gradients in fire severity (biomass combustion and residual vegetation) and secondarily to gradients in soil pH and regional climate. This pattern was apparent in both the full dataset (n = 87 sites) and for a reduced subset of sites (n = 49) that minimized the correlation between site moisture and fire severity. Changes in community composition across the fire-severity gradient in Alaska were strongly correlated to variations in plant regeneration strategy and rooting depth. The tight coupling of fire severity with regeneration traits and vegetation composition after fire supports the hypothesis that disturbance characteristics influence patterns of community assembly by affecting the relative success of different regeneration strategies. This study further demonstrated that variations in disturbance characteristics can dominate over environmental constraints in determining early patterns of community assembly. By affecting the success of regeneration traits, changes in fire regime directly shape the outcomes of community assembly, and thus may override the effects of slower environmental change on boreal forest composition.

## Introduction

The structure and composition of plant communities is governed by a hierarchy of abiotic and biotic filters that act on the regional species pool to control which species and traits are present at a site [1], [2]. Both site conditions and stochastic processes, such as disturbance and propagule dispersal, influence temporal and spatial heterogeneity of community assembly [3]. Measures of community composition and diversity are often used to infer the responses of community assembly to environmental gradients or other factors. These community indices provide strong evidence about *which* species occur in a given time or place. A focus on plant traits can provide additional insights into community assembly by indicating *why* certain species are arrayed along observed gradients [4]. Patterns and processes underlying the expression of plant traits on the landscape are important to ecosystem functioning but are poorly understood in many environments [4], [5].

Variation in the distribution and severity of fire disturbance influences landscape and stand-level vegetation patterns (e.g. [6], [7], [8]), in part through the influence of plant regeneration traits on patterns and processes of community assembly [9]. In systems with recurring fire, disturbance acts as a filter to select species with regeneration traits adapted to that particular fire regime. Community assemblages can therefore be altered when a fire regime changes in attributes such as frequency or severity [10].

Fire is the dominant disturbance in the North American boreal forest, as reflected in regional and local species pools and landscape-level vegetation patterns [6], [11]. Fire frequency increased in the Alaskan boreal forest about 7,000 years ago as black spruce spread across the landscape in response to a moister, cooler climate [12]. Current landscape variation in species composition is driven by interactions between fire characteristics (e.g. severity, size, and frequency), site characteristics (e.g. site moisture and drainage, glacial history, and local climate) and plant regeneration traits [13], [14]. However, distinguishing between traits that are adaptations to fire or to some other factor, such as cool climate or site moisture, is not always possible [10]. Disentangling the effects of fast-changing factors such as fire regime versus more slowly changing factors is an essential step in predicting the initial responses of ecosystems to rapid environmental change [15].

During the 20^th^-century, boreal Alaska has warmed twice as rapidly as the global average, with warming since the 1950s apparently unprecedented in at least the last 400 years [16]. Warming in Alaska has been associated with a shift in the fire regime to larger and more frequent fires [17], a trend that is projected to continue [18]. Increases in large fire years and late season burning have stimulated an increase biomass consumption, or fire severity [19]. This increase in fire severity has been associated with altered community composition in black spruce forests of boreal Alaska [14], [20]. However, it is unknown whether these shifts in composition are simply the net sum of individualistic species responses to environmental change, or represent a more predictable shift in the abundance of species with regeneration traits favored by a new set of fire characteristics [1], [21].

We measured the composition of early post-fire vegetation communities following the 2004 fire season, the largest annual area burned in Alaska’s 58-year fire record [22]. We hypothesized that, if disturbance characteristics influence post-fire community assembly, variations in fire conditions should be reflected in patterns of post-fire community composition. More specifically, we predicted that variations in fire severity would affect community composition by altering the relative success of plant regeneration strategies after fire. We tested these hypotheses by a) comparing patterns of post-fire vegetation composition across a range of fire severities observed within a single pre-fire forest type, and b) linking fire characteristics to the relative abundance of plant regeneration strategies at the species and community level. The study sites also spanned a broad gradient in moisture availability, the major environmental determinant of post-fire recruitment [14], enabling us to assess the relative importance of fire severity and environment in influencing post-fire community assembly. Our results provide an empirical test of the mechanisms by which disturbance characteristics can shape patterns of community assembly in post-fire secondary succession.

## Materials and Methods

### Study Area

**Figure 1:**
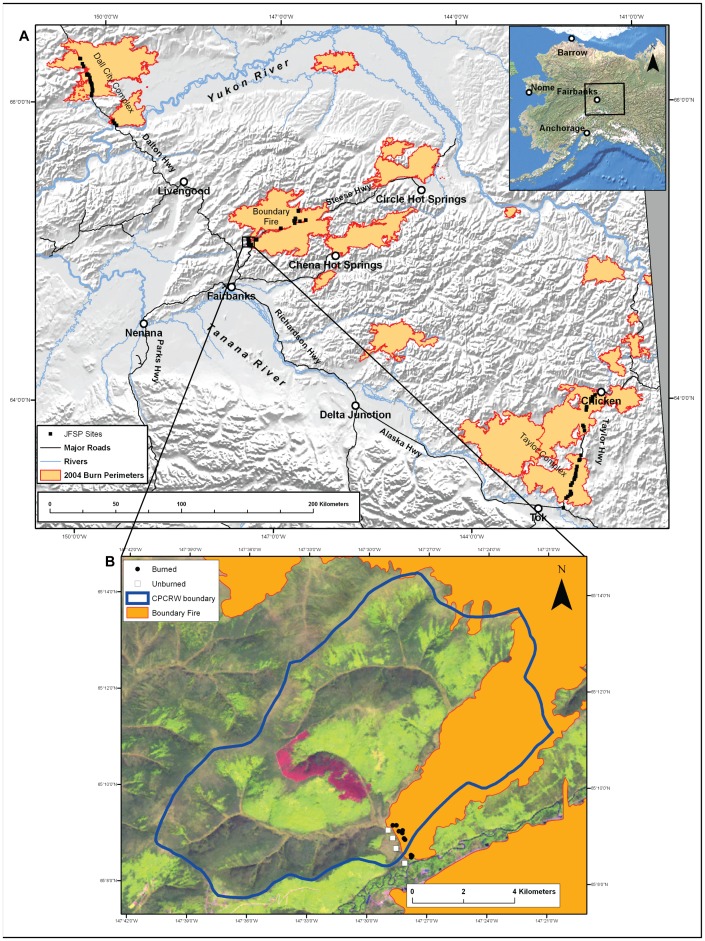
The location and extent of the study area. (a) Within Alaska (inset) and across 3 burn complexes, showing the sampled sites from 2004 burns (n = 87) (modified from [14]) and (b) a higher resolution map of Bonanza Creek LTER Caribou/Poker Creeks Research Watershed (CPCRW) where unburned (n = 4) and burned plots (n = 8) were sampled for species rooting depth measurements.

Interior Alaska is a region of extensive but discontinuous permafrost, with a continental climate and temperature extremes ranging from −70°C to +35°C [23]. Our study area is characterized by small mountain ranges, gently sloping uplands, and large areas of lowlands (Fig.1). Black spruce forests are the dominant forest type of Interior Alaska; occupying approximately 45% of the forest landscape [24] and covering north-facing slopes, dry ridge tops, and poorly drained forested lowlands. Low moisture content of plant leaves and high flammability of needles and surface fuels make black spruce forests highly susceptible to fire disturbance [24].

Widespread fires in 2004 burned 2.7 million ha of forest in Interior Alaska [22]. We established study sites in May 2005, immediately after snowmelt in the year after the fires. These sites were dispersed across Interior Alaska along three road networks that intersected three burn complexes, each with multiple ignition points: The Taylor Highway (Taylor Complex), the Steese Highway (Boundary Fire), and the Dalton Highway (Dall City Complex) (Fig. 1a). For accessibility, sites were selected within walking distance of a road, but beyond any signs of disturbance associated with the road corridors, which were often gravel roads crossing through otherwise undeveloped lands (distances of 100–600 m from the road).

### Field Measurements

Following an initial reconnaissance of the burned areas, we selectively identified 90 sites (∼30 sites in each burn complex) that encompassed the range of fire severities, site moisture, topographic positions, and geographic dispersion that we encountered in burned black spruce forests [14]. Of the original 90 sites, we removed one site that was a persistent outlier in all our community analysis, a second that was not sampled for vegetation, and a third site that had only partial fire-severity data, for a final total of 87 sites. All sites were dominated by black spruce when they burned. Pre-fire understory communities included moist and dry, acidic and non-acidic communities as well as elevational treeline communities [25]. Most of the sites (82 of 87) burned with ≥95% mortality of the pre-fire trees. Our study sites were located in Bureau of Land Management (BLM), State of Alaska (SoA), and University of Alaska land. Permits and approval for sampling post-fire vegetation were granted from both BLM (Lenore Heppler- field manager for the Eastern Interior Field Office, casual use permit) and SoA (Director of the Division of Mining, Land, and Water- Department of Natural Resources). Permission was also obtained through the Bonanza Creek Ecological Research program (BNZ LTER).

In 2005 and 2006 we measured environmental conditions, pre-fire stand composition, and fire characteristics at each site, as described in detail elsewhere [14], [20], [26]. Environmental conditions included elevation, latitude and longitude, slope and aspect, soil pH, and a 6-point index of potential site moisture ranging from xeric to sub-hygric [14] that was then grouped into three moisture classes (moist, mesic, and dry). Spot soil moisture measurements were taken with a hand-held moisture probe (Hydro-Sense, Campbell Scientific, Edmonton, AB, Canada) inserted into the upper 10 cm of mineral soil at each site (n = 10/site) during a three-week period in July 2006 and confirmed the general pattern of site moisture classifications (r = 0.77). Pre-fire stand characteristics were quantified as the density and basal area by species of all trees >1.4 m height and rooted within a 120 m^2^ sub-sample area at each site [26]. Pre-fire stand age was estimated as the age of the oldest tree based on ring counts from basal stem samples of 5 canopy dominant trees/site [14]. Fire severity was quantified based on three metrics: the Composite Burn Index (CBI; [27]), the depth of the residual organic layer, and the proportion of the organic layer burned away by the fire as estimated from adventitious roots on pre-fire black spruce [26]. As CBI was significantly correlated with both residual organic layer depth [14] and organic layer consumption [26], we used thresholds in CBI derived from tree seedling responses [14] to separate sites into classes of “high”, “moderate”, and “low” severity fire (CBI values ≥2.5, between 2.5 and 2, or ≤2; n = 32, 33, and 22 sites, respectively).

Measurements of post-fire plant community composition were made at each site in July 2006 to quantify the initial responses of the plant community to the 2004 wildfires. We conducted a relevé at each site following the methods of [25]. The relevé consisted of a list of all vascular and nonvascular plant species present and an estimate of percent cover of each species using a modified Braun-Blanquet cover-abundance scale [20] (0.1 = rare, 0.5 = common but less than 1%, 1 = 1−2%, 2 = 3−5%, 3 = 6−10%, 4 = 11−25%, 5 = 25−50%, 6 = 51−75%, and 7 = 75−100% cover). Vascular plant nomenclature follows the Panarctic Flora checklist [28] except for species missing from the checklist, where nomenclature follows Missouri Botanical Garden’s TROPICOS nomenclature database (http://www.tropicos.org). Nomenclature for lichens follows [29] and bryophytes generally follow TROPICOS. We determined the regeneration strategy for each species at a site based on visual evidence and excavation of plant individuals as needed. Every species at a site was classed as residual (did not burn in the fire and remained alive after fire), resprouter (burned in the fire and then resprouted from stems or roots), or colonizer (recruited from seed via dispersal or the local seedbank). If a species occurred in two different regeneration modes at a site, especially common in *Salix* spp., the relative proportion of individuals arising from each regeneration mode was estimated based on plant morphology and size.

To estimate the potential of plant species to survive a fire via underground organs, we documented the rooting depth and root biomass distribution of species in eight burned and four unburned plots (hereafter “rooting plots”). Plots were selected to span a range of topographic positions representative of black spruce forests, and were located in the Caribou Poker Creeks Research Watershed (CPCRW) about 45 km northeast of Fairbanks (Fig. 1b). CPCRW is part of the BNZ LTER, which partially burned in the 2004 Boundary Fire. Each plot was located within an area of at least 50×50 m that appeared to be largely homogenous in stand structure, site moisture, and, if appropriate, fire severity.

We measured belowground distribution of root and rhizome biomass of all vascular species found along a randomly-oriented 30 m×5 m transect in each of the four unburned plots (n = 34 vascular species total). At 5 m intervals, we measured the closest individual of all vascular species until 5 individuals of each species were obtained in each plot (n = 5/species/rooting plot). Belowground measurements were taken by carefully digging up the rooting structure of the individual until the lowest depth of secondary roots and rhizomes was found. For each individual we collected all belowground biomass of the rooting structure and separated it by the soil layer in which it was found. Soil layers were categorized as moss (living moss), upper duff (organic matter that is still identifiable), lower duff (unidentifiable and well-decomposed organic matter), and mineral soil. Belowground biomass was then dried and weighed in the laboratory. For three obligate post-fire species (*Corydalis sempervirens, Equisetum sylvaticum,* and *Luzula rufescens)* not found in our unburned rooting plots, we made similar measurements in the eight burned plots within 1 km of our unburned rooting plots (Fig. 1b). All data were deposited in the BNZ LTER database (http://www.lter.uaf.edu/data_b.cfm).

### Data Analysis

#### Trait selection

Studies that relate regeneration traits to community composition across multiple sites typically do so by either assigning a binary response to a given species across all sites (e.g. sprouting versus non-sprouting [9], [30]); or assume a constant value for a continuously distributed trait from literature values (e.g. [31], [8]). However, because trait values of individual species may vary phenotypically across the landscape, we measured and quantified variation in post-fire regeneration traits for every species we encountered in our study area (n = 81 species).

We calculated the frequency of occurrence of dominant regeneration modes (colonizer, resprouter, residual; e.g. [31]) for each species across all 87 sites. We further classified species by their predominant rooting layer (moss, upper duff, lower duff, and mineral soil) using measurements of the relative distribution of belowground biomass. This metric is intended to characterize the potential effect of different levels of soil combustion on plant resprouting ability from belowground plant parts [32]. Although we found some plant-level and site-level variations in root distributions within species, our sample size was insufficient to determine the cause of this variation. Thus, it was necessary to collapse variability in this trait into single values that could be used to represent species across all our sites. For the 28 understory vascular species for which we had field measurements of belowground biomass, we calculated the relative weight of biomass located within each rooting substrate and averaged this across the 4 unburned or 8 burned rooting plots. For tree species, we estimated belowground biomass distributions based on our previous observations of substrate layers that could support seedling establishment [33], [14]. For mosses, we characterized the soil substrate in which the green portion of the moss was anchored using two assumptions: a) moss species that occurred only as residuals on patches of undisturbed organic layer were assumed to “root” entirely in the moss substrate, and b) mosses species that occurred as post-disturbance colonizers (*Ceratodon purpureus*, *Marchantia polymorpha*, *Polytrichum commune*, and *Polytrichum strictum*) were characterized based on our qualitative observations of the frequency with which we encountered them on different substrates across all 87 sites. For example, it was extremely rare to observe *Ceratodon purpureus* on any other substrate besides mineral soil, whereas *Polytrichum* spp. were observed to be anchored on both lower duff and mineral soil. Finally, all lichens observed after fire were residual lichens anchored in the unburned organic layer and were categorized as “rooting” in the moss substrate. Vascular species for which we did not have data on belowground biomass distribution (22 of the 81 understory species) were given zero values for all rooting substrates, so that these species provided no information on the distribution of this trait.

#### Deriving the distribution and abundance of traits across sites

We based our multivariate analyses of plant responses to fire disturbance on three matrices of primary data and one derived matrix. Matrix nomenclature follows [34]. The main matrix (A) contained abundance data for all vascular and non-vascular plant species in the form of Braun-Blanquet cover classes (87 sites by 81 species). A second site matrix (E) contained data on environmental conditions, pre-fire stand composition, and fire severity (87 sites by 21 variables). Finally, a third matrix (S) contained information on plant traits for each species (7 traits by 81 species). Trait columns in this matrix included the frequency of observed regeneration modes (colonizer, resprouter, residual) and the proportional distribution of belowground biomass in moss, upper duff, lower duff, and mineral soil rooting substrates. We calculated a fourth, derived data matrix (AS’) by multiplying the A and S matrices to obtain a distribution of species traits across sites (87 sites by 7 traits) that was weighted by species abundance (Table S1). After multiplying the matrices, data on regeneration modes and rooting substrates were both relativized, so that trait values for regeneration mode or for rooting substrate each summed to one for a given site.

#### Variation in species composition and plant traits across sites

We determined the species and family richness of our post-fire flora. We calculated alpha diversity as the average species richness per site and beta diversity as the ratio of the total number of species across all sites to the average number of species per site [34]. We performed ANOVAs (SPSS Inc. Released 2008. SPSS Statistics for MacIntosh, Version 18.) to test for differences in species richness between fire severity levels. Single occurrence species, and two species that occurred twice were then removed for subsequent analyses.

We performed Nonmetric Multidimensional Scaling (NMDS) ordinations [35] on the A matrix (PC-ORD for Windows. Multivariate Analysis of Ecological Data, Version 6) to describe and quantify patterns in post-fire species composition across sites and between fire severity classes. We used the Sorensen (Bray-Curtis) [34] distance measure to represent compositional dissimilarity. The final dimensionality (i.e. number of axes) was determined by comparing the stress values against dimensionality to identify a solution with the lowest stress relative to the number of axes [34]. We correlated three matrices with NMDS site scores: 1) the A matrix, 2) the E matrix and 3) the AS’ matrix and expressed high correlations (r≥0.25) with a biplot overlay to indicate both the direction and magnitude of the strongest secondary variables in the ordination space. Finally, we calculated a post-hoc proportion of variance represented by each axis by calculating the squared Pearson correlation (r^2^) between pair-wise distances of objects in ordination space and distances in the original distance metric [34].

Our initial ordination results indicated that plant responses to moisture and fire severity across all 87 sites were complicated by a confounding association between pre-fire and post-fire organic layer depths that reflected a broad constraint of site moisture on fire severity [26], [36]. To better disentangle the effects of site moisture and fire severity on post-fire community composition, we identified a subset of 49 sites that minimized the correlation between pre-fire and post-fire organic depths within a given burn or moisture class. Variations in fire severity at these sites were likely tied to variations in fire weather and seasonal thaw at the time of burning [36], rather than being dominated by pre-fire differences in site moisture. We performed NMDS ordination on the subset of 49 sites following the procedures described above for the full dataset.

#### Variation in post-fire plant traits across species and growth forms

To evaluate the influence of fire on the relative distribution of plant regeneration traits, we compared the frequency of traits between the post-fire species pool and the observed trait distribution at each site [21]. The observed trait distribution was calculated as average values of relativized traits from the AS’ matrix described above. Thus, these values represent trait frequencies weighted by the relative abundance of species at a site. We calculated average traits for three groups of sites representing high, moderate, and low severity fires (see ***field measurements***).

We calculated the % frequency of regeneration strategy and rooting depth across species as well as within plant growth forms (deciduous trees/shrubs, forbs, evergreen trees/shrubs, graminoids, lichens, mosses) and between fire severity classes. Finally, to determine the likelihood of a given regeneration strategy occurring on a given substrate, we closely examined species that exhibited multiple regeneration strategies across sites. We built a multinomial logistic regression model to test whether likelihood of a particular regeneration strategy was correlated to % of a site covered in organics as well % of the site covered in <3 cm organic depth (SPSS Inc. Released 2008. SPSS Statistics for MacIntosh, Version 18.0). The 3 cm threshold in organic depth is an experimentally determined threshold for germination of deciduous tree species [33] that has been linked to post-fire understory species composition [20].

## Results

### Variation in Post-fire Species Composition and Plant Traits Across Sites

Across all 87 post-fire sites, we encountered 134 plant and lichen species distributed across 50 families (Table S2). We observed 101 vascular plants (35 families), 18 bryophytes (9 families, and 7 *Sphagnum* spp.), and 15 lichens (6 families). Of the 101 vascular plants, there were 4 trees (out of 6 tree species found in Interior Alaska), 43 forbs, 17 graminoids, 18 deciduous shrubs, 12 evergreen shrubs, and 7 seedless vascular plants. This is approximately 50% fewer plant species than found in mature black spruce stands (30% fewer vascular and 75% fewer nonvascular species) [25]. Across all 87 sites (each 2500 m^2^), alpha diversity (average species richness/site) was 19.93±0.51, beta diversity was 6.08 (the ratio of total number of species across all sites to average number of species at a site), and gamma diversity was 134 (total number of species across all sites). Species richness varied significantly among fire severity classes (ANOVA F = 11.37, p<0.001), with richness declining as fire severity increased. Two years after burning, low-severity sites had an average richness of 25.34±1.09 species per site (mean ± SE), significantly greater than sites with moderate severity (21.18±0.94 species per site, Tukey post-hoc p<0.001) or high severity (18.50±0.77 species per site; Tukey post-hoc p<0.001).

**Figure 2:**
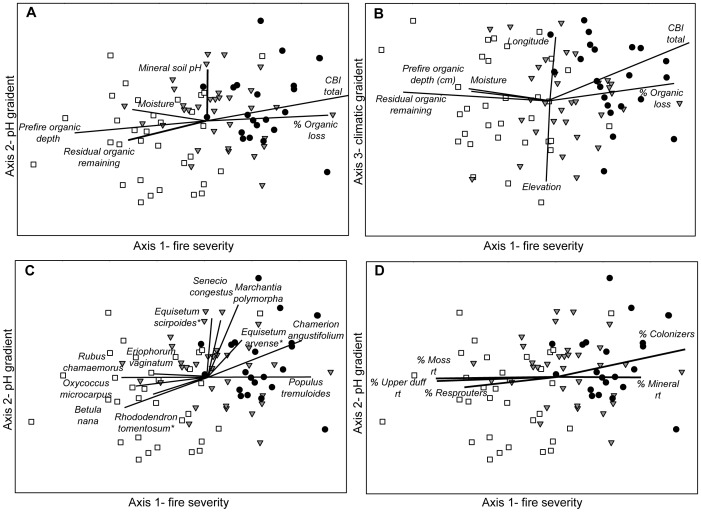
NMDS ordinations of all sites. Sites are grouped by fire severity (• = high severity sites, ▾ = moderate severity, and □ = low severity sites), showing Axis 1 versus Axis 2 (a, c, d) and Axis 1 versus Axis 3 (b). Strong correlations (r>0.25) with the ordination axes are indicated by biplot vector where length and direction represent the magnitude and direction of correlation, respectively. Ordination axes were correlated with abiotic and biotic environmental variables (a,b), species composition (c), and plant trait composition (d). Axis 1 represents a fire severity gradient, Axis 2 represents an acidity gradient, and Axis 3 represents a climatic gradient. For ease of interpretation, all vectors are shown at 150% magnitude.

Ordination of sites in species-space (Matrix A) captured three dominant gradients (axes) in species composition (Fig. 2). The NMDS ordination had a final stress of 16.60, indicating that the ordination provided a reasonable representation of species composition patterns [34]. The three NMDS axes captured 79.2% of the variance in post-fire species composition across all sites and the final ordination solution was highly stable, with an instability of 0.0025 after 500 iterations.

All three axes of the NMDS ordination were strongly correlated with gradients in measured environmental factors (Fig. 2). The strongest axis in the ordination (representing 42.2% of the variance in the original distance matrix) was highly correlated with fire severity metrics such as CBI (composite burn index), percent loss of the soil organic layer, and depth of the residual organic layer remaining after fire (Table 1). This “fire severity axis” also showed strong correlations with site moisture and pre-fire organic layer depth (Fig. 2a, b), likely representing the combined response of vegetation to site moisture, pre-fire organic layer depth, and fire severity. The second major ordination axis (20.3% of the variance) was correlated with the pH of the mineral soil (Table 1, Fig. 2a). Gradients in species composition that were associated with mineral soil pH were orthogonal to variations associated with fire severity (Fig. 2a), indicating that pH appeared to influence vegetation patterns relatively independent of soil moisture and fire severity across the study sites. The third major axis of the ordination (16.8% of the variance) was related to gradients in elevation and longitude (Table 1, Fig. 2b). These spatial gradients were also largely independent of the primary gradient in fire severity and likely represented the effects of large-scale spatial variations in environmental conditions (such as local climate) on plant communities.

**Table 1:** The Kendall correlations of species, site characteristics, and plant traits for NMDS Axes 1, 2, and 3.

Species	Axis 1	Axis 2	Axis 3
*Betula nana*	−**0.494**	−0.302	−0.235
*Ceratodon purpureus*	0.31	**0.447**	0.351
*Corydalis sempervirens*	**0.491**	−0.132	0.071
*Chamerion angustifolium*	**0.658**	**0.448**	0.264
*Equisetum arvense* *	0.403	**0.463**	0.047
*Equisetum scirpoides* *	0.202	**0.534**	−0.338
*Equisetum sylvaticum*	−0.09	−0.128	**0.589**
*Eriophorum vaginatum*	−**0.502**	0.013	0.193
*Rhododendron tomentosum* *	−**0.567**	−0.383	0.308
*Marchantia polymorpha*	0.391	**0.615**	0.268
*Oxycoccus microcarpus*	−**0.553**	−0.221	0.369
*Pleurozium schreberi*	−**0.45**	−0.282	−0.139
*Aconogonon alaskanum*	−0.133	−**0.472**	0.237
*Populus tremuloides*	**0.654**	0.004	0.218
*Rubus chamaemorus*	−**0.581**	−0.114	0.331
*Senecio congestus*	0.282	**0.551**	0.167
*Sphagnum girgensohnii* *	−**0.471**	−0.098	0.034
*Vaccinium uliginosum*	−**0.485**	−0.185	−0.054
**Site characteristics**			
Elevation (m)	−0.184	−0.015	−**0.584**
Latitude (N)	0.051	−0.158	**0.46**
Longitude (W)	0.082	−0.137	**0.522**
pH	−0.12	−**0.658**	−0.177
Site moisture	−**0.582**	0.242	0.137
total CBI score	**0.761**	0.345	0.287
Residual organic remaining	−**0.782**	−0.238	−0.042
Prefire organic depth	−**0.606**	−0.338	0.172
Organic layer lost	**0.733**	0.15	0.165
**Traits**			
% regenerators	−**0.716**	−0.234	−**0.558**
%colonizers	**0.753**	0.377	**0.448**
% residuals	−0.125	−0.168	0.292
% moss rooters	−**0.741**	0.015	−0.323
% upper duff rooters	−**0.569**	0.035	0.036
% mineral rooters	**0.63**	0.015	0.326

* Diagnostic species for black spruce communities (Hollingsworth et al. 2006).

**Bold** correlations have an r^2^>.2.

We assessed the correlation of ordination axes with the main species (A) matrix and a plant trait matrix (AS’). Many species that are indicators of a high or low severity fire were significantly correlated with Axis 1 (Fig. 2c, Table 1). For example, the tree *Populus tremuloides* and forb *Chamerion angustifolium* were most commonly found in severely burned sites, while shrubs *Betula nana* and *Rubus chamaemorus* were positively associated with sites that burned at low severity. Among the species that showed strong correlations with Axis 2 (Table 1, Fig. 2c), two (*Equisetum arvense* and *Equisetum scirpoides*) have been characterized as diagnostic species for nonacidic mature black spruce communities and a third species (*Rhododendron tomentosum*) is diagnostic of acidic black spruce communities [25]. Only one of 134 species (*Equisetum sylvaticum*) in our study showed a strong correlation (r^2^>0.2) with axis 3 (climate).

Variations in post-fire plant community composition were also strongly related to the weighted abundance of different plant traits, as represented by our derived site-by-trait (AS’) matrix. Associations with different regeneration modes and rooting substrates were predominantly expressed along the fire severity gradient represented by Axis 1 (Table 1, Fig. 2d). Colonizers and species that root in mineral soil were more abundant after severe burns, while resprouters and species that rooted in the upper duff and moss layer were most abundant after low severity burns (Fig. 2d).

### Assessing Confounding Effects of Site Moisture and Fire Severity

**Figure 3:**
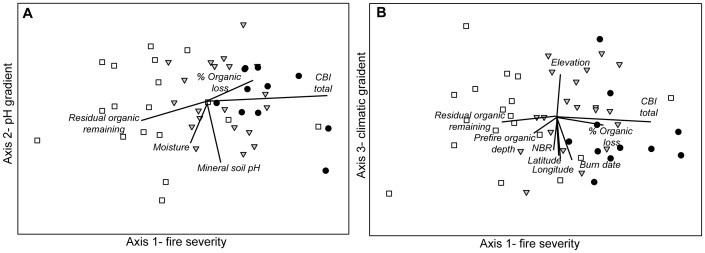
NMDS ordinations of a subset of sites. This subset had low correlation between site moisture and fire severity. Sites are grouped by fire severity (• = high severity sites, ▾ = moderate severity, and □ = low severity sites), showing Axis 1 versus Axis 2 (a) and Axis 1 versus Axis 3 (b). Ordinations were correlated with abiotic and biotic environmental variables for comparison with Fig 2 a,b. Strong correlations (r>0.25) with the ordination axes are indicated by biplot vector. As with the ordination of the full set of sites, in the subset of sites Axis 1 represents a fire severity gradient, Axis 2 represents an acidity gradient, and Axis 3 represents a climatic gradient.

A chi-squared contingency table analysis (Table 2) of our 87 sites showed a significant relationship between site moisture and fire severity (χ^2^ = 8.99, p = 0.0112, df = 2). It is therefore difficult to know if the correlation of plant traits with axis 1 is a consequence of site moisture or fire severity. To better disentangle the effects of site moisture and fire severity on post-fire community composition, we analyzed patterns of species composition across a subset of sites with minimized correlation between pre-fire and post-fire organic depth. In this subset of sites, observed volumetric soil moisture explained 8% of the variance in site-level CBI (F = 4.01, df = 47, p = 0.05), a substantially weaker association than observed between observed volumetric moisture and CBI in the full dataset (r^2^ = 0.14, F = 13.29, df = 82, p = 0.0005). We found very similar ordination patterns with these sites as we did with the full number of study sites (Fig. 2a,b, versus Fig. 3a,b). The NMDS species ordination of the subset of sites resulted in a three-dimensional solution with a final stress of 16.99, and the final instability of the ordination solution was 0.0001 after 500 iterations. The three major gradients captured 75.9% of the variance in post-fire species composition. Correlations with environmental variables indicated that Axis 1 represented a fire severity gradient (36.5% variance), Axis 2 corresponded to a soil acidity gradient (22.3% variance), and Axis 3 represented a spatial climatic gradient (17.1% variance) (Fig. 3a,b). Although the ordination patterns and correlations of environmental variables and trait abundances were similar between the reduced and full dataset, ordination of the reduced dataset showed no significant axis correlations with site moisture. This suggests that the variation in plant traits observed post-fire was primarily related to variation in fire severity, as plant traits were distributed along gradients in fire severity even in the absence of a detectable effect of soil moisture.

**Table 2:** Chi-squared contingency table to evaluate whether there is a significant difference in distribution of sites based on moisture and severity.

	Site moisture (treatment)		
Fire severity (outcome)	Low	High	Total number of sites
**Low**	15	17	32
**Medium**	15	11	33
**High**	22	3	22
***Total number of sites***	*56*	*31*	***87***

### Variation in Plant Traits Across Species and Growth Forms

**Figure 4:**
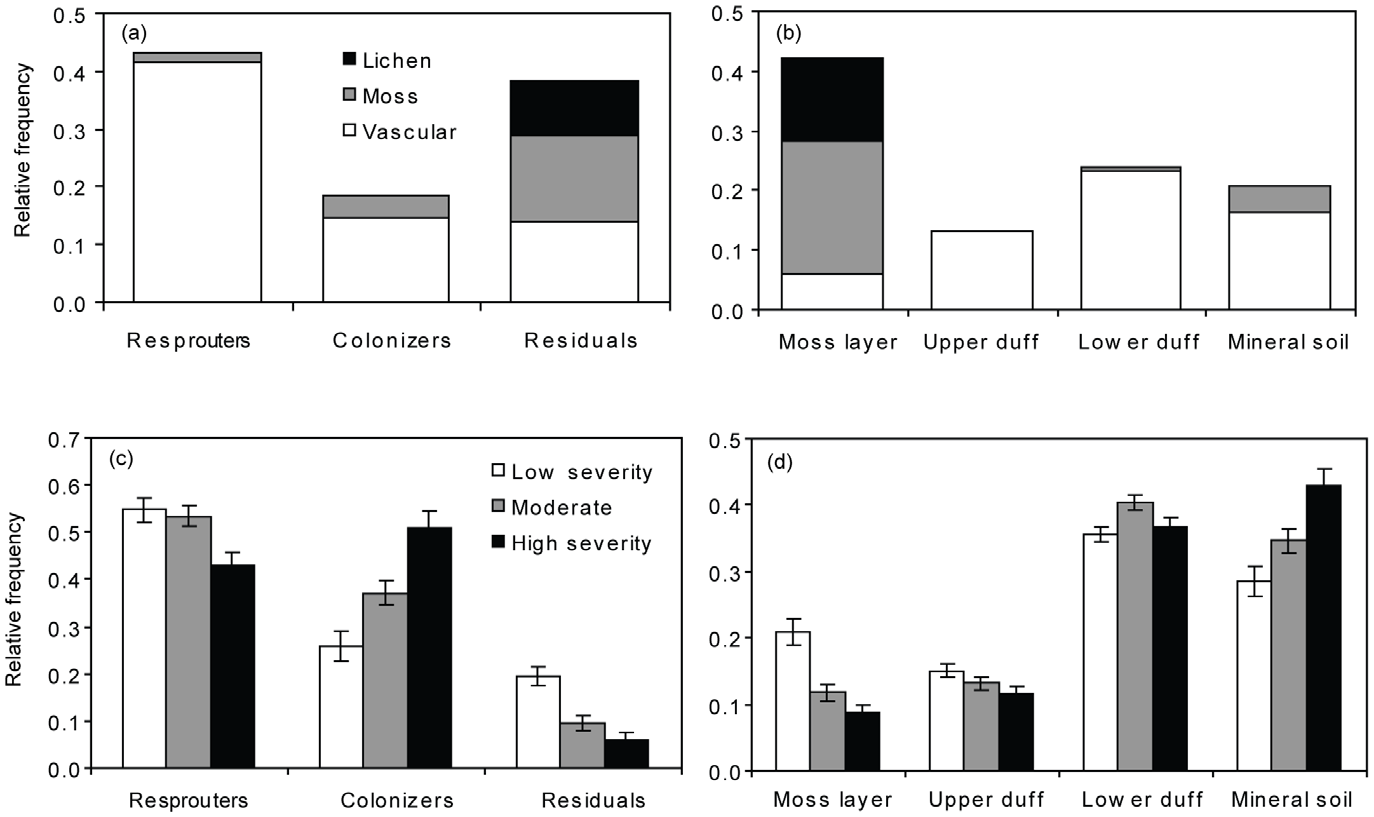
Histograms depicting the distribution of plant traits. Distributions range across the regional species pool of post-fire black spruce communities (a,b) and the realized, abundance-weighted trait distribution in post-fire communities that experience different levels of fire severity (c,d). Two sets of traits related to post-fire regeneration are shown: (a,c) Regeneration mode separated into categories of regenerators, colonizers, and residuals, and (b,d) Distribution of belowground biomass across different rooting substrates (moss, upper duff, lower duff, and mineral soil). For the regional species pool, the distribution of traits has been separated into understory growth forms of lichens, mosses, and vascular plants.

Over 40% of the post-fire species displayed the “resprouter” strategy, making it the most common regeneration mode in the post-fire species pool (Fig. 4a). This asexual regeneration strategy is commonly associated with vascular plants with woody stems or belowground rhizomes. However, a few mosses (notably *Sphagnum* spp.) also exhibited this strategy, as they are able to resprout from points along the stem below the capitulum [37]. A large portion of species encountered in the post-fire communities were also associated with the “residual” strategy, in which species become part of the post-fire community by surviving in patches of unburned vegetation (Fig. 4a). Although most of the stands we sampled had >95% mortality of the overstory trees, many of these stands still contained patches of unburned surface vegetation. These patches provided an important source of post-fire plant diversity, accounting for most bryophyte species and all lichen species in these early post-fire communities. Because unburned patches were commonly located around moist microsites, such as hummocks of sphagnum moss or moist hollows, the residual strategy may represent a secondary effect of species sorting along moisture gradients. Finally, approximately 20% of the post-fire species encountered were classified as colonizers arising from seed (Fig. 4a). The colonizer group included many of the “weedy” post-fire species frequently encountered in recently disturbed areas. There were also several *Salix* spp. that recruited from seed and are a particularly important component of the colonizer group because they often persist through succession to mature stands [25]. Species differed in their distribution across rooting substrates (Fig. 4b). Most bryophyte species and all lichen species in the post-fire species pool were characterized as being “rooted” or anchored in the moss layer. Many of the vascular plant species had belowground biomass in the lower duff and mineral soil layers, giving rise to a second peak in the vertical distribution of belowground biomass (Fig. 4b).

The effect of fire severity as a filter on the distribution of plant regeneration traits can be visualized by comparing the observed, abundance-weighted distribution of regeneration traits in different severity classes (Fig. 4a,b) versus the distribution of traits among species in the entire post-fire species pool (Fig. 4c,d). If there were no sorting effects of fire, abundance-weighted distributions of species should reflect the distribution of traits across species in the post-fire pool. In general, fire reduced the abundance of “residuals” compared to their proportional number in the post-fire species pool, and this reduction was greatest at high-severity sites, where unburned patches were less common (Fig. 4c). In contrast, the relative abundance of “colonizers” was higher in burned sites compared to their proportional number in the post-fire species pool; this increase was greatest at high-severity sites. Species using the “resprouter” strategy were most common at low- and moderate-severity sites and “residuals” were also most abundant at low-severity sites. For rooting traits, species rooted in the moss layer were less abundant after fire than their distribution in the species pool would predict. In contrast, species rooting in the lower duff and mineral soil showed a higher than expected proportional abundance after fire (Fig. 4d).

Although most post-fire species encountered were characterized by a single regeneration strategy across all sites, eight of the species exhibited site-dependent regeneration strategies. Two evergreen shrubs (*Rhododendron tomentosum* and *Vaccinium vitis-idaea*) exhibited both resprouter and residual strategies, three deciduous trees/shrubs and two forbs (*Betula neoalaskana*, *Salix scouleriana*, *Arctous rubra*, *Equisetum scirpoides*, and *Equisetum sylvaticum*) exhibited both resprouter and colonizer strategies, and one moss (*Polytrichum commune*) exhibited all three regeneration strategies. However, multinomial logistic regressions predicting the likelihood of a given regeneration strategy for a given species from site substrate characteristics were not significant (p>0.1).

## Discussion

We found clear evidence of disturbance characteristics shaping post-fire community assembly in Alaskan boreal forests. Observed associations between post-fire community composition and disturbance characteristics, environmental variables, and plant traits are consistent with theoretical predictions of a hierarchy of factors controlling community assembly [4]. In particular, this study provides evidence of two main filters on plant community assembly in post-fire boreal forests: a) impacts of fire characteristics on the relative success of different regeneration strategies, with consequent impacts on species abundance in the post-fire species pool, and b) effects of slowly changing environmental factors such as pH on species abundance independent of regeneration traits.

Variations in fire severity observed across our network of 87 post-fire sites were strongly linked to patterns of post-fire community composition, and this pattern persisted even when we reduced the confounding effects of site moisture on fire severity by examining a subset of sites. This is consistent with concurrent findings from a subsample of these sites, where specific comparisons of pre- and post-fire vegetation found a dominant effect of fire severity on observed changes in species composition [20]. Post-fire plant communities have been demonstrated to be sensitive to variations in fire characteristics across a wide range of boreal forests (e.g. [31], [38], [39]). The novel aspect of this study is the evidence from a large number of forest sites that the link between fire characteristics and plant communities is mediated by plant regeneration traits. The close alignment we observed between fire severity gradients and plant trait abundance is consistent with the hypothesis that post-fire plant regeneration is tied to fire severity via effects on the relative success of different regeneration traits. If regeneration traits do not shape patterns of community assembly, we would expect to see little or no correlation between trait abundances and community composition. Instead, we found strong associations both between trait patterns and community composition, and between regeneration traits and fire severity. These results suggest community responses to changes in fire characteristics are predictable from a set of underlying mechanisms that link regional species pools and fire characteristics to post-fire outcomes via measurable regeneration traits (e.g. [1], [9]).

Research in the boreal forest has linked community composition to environmental factors. Across our study area (e.g. road accessible Interior Alaska), community composition and structure in unburned, mature black spruce has been strongly linked to site moisture, mineral soil pH and elevation [13], [25]. Our results also illustrate the importance of these environmental variables in shaping early post-fire vegetation. However, the results of this study provide two noteworthy results with respect to interpreting factors controlling community assembly in boreal forests. Firstly, dominant patterns of variation in post-fire community composition were primarily correlated with fire severity and only secondarily with abiotic factors such as site moisture, soil acidity and climate. Thus, our results show that variations in fire characteristics can play a dominant role in determining community composition, even within a system known to be highly structured by environmental gradients. Secondly, relationships between plant community composition and environmental gradients were largely *independent* of plant regeneration traits. This is in marked contrast to the association between fire severity gradients and regeneration traits and suggests that plant responses to environmental gradients *other* than fire severity are governed by traits not measured in this study.

Nevertheless, variations in fire characteristics are strongly linked to environmental gradients, and we found significant associations between fire severity and site moisture across our study sites. Paleo-records suggest that this relationship has existed in boreal Alaska for as long as black spruce has dominated the landscape [12], [40]. This makes it difficult to tease apart the relationship between plant community composition, and subsequently plant traits, and fire severity [10]. Because of our large dataset, however, we are able to further assess this relationship. By restricting our analysis to a large subset (49 sites) of sites with minimal correlation of pre and post-fire organic depth, we removed most of the confounding effects of site moisture on fire severity and still found a nearly identical species compositional pattern as with our full set of sites. Most importantly, our subset of sites showed that variation in species composition and plant traits were related to the same corresponding environmental gradients (fire severity, acidity, and climate). This additional evidence strongly suggests that fire severity acts as a primary filter on plant regeneration traits and subsequent community assembly, and therefore has the potential to overcome community legacies associated with slowly changing variables such as pH or climate.

Observational results suggested that a few species had multiple regeneration strategies and therefore were able to regenerate across a broader range of fire severities. We were interested in whether we could use species with multiple regeneration strategies to test the importance of site substrate characteristics to a given regeneration strategy, or whether a species may exhibit preferential use of a regeneration strategy depending on substrate characteristics. Our inability to statistically predict regeneration strategy of a species at a given site was likely due to the low number of observations (between 8–14 sites, depending on the species), however, poor prediction of regeneration strategy from substrate characteristics could also suggest that species with multiple regeneration strategies are responding to other environmental factors that act as filters on boreal forest composition [13], [25].

Regeneration modes that were important in Alaska apply to many fire-prone ecosystems [8], [9], [30], [31]. In boreal forests, the success of seed colonizers is particularly tied to substrate characteristics, and resprouters often use asexual reproduction from belowground roots and shoots as a primary means of regeneration. Fire consumption of thick organic layers provides a better substrate for seed germination and seedling survival, which is particularly important in providing colonization opportunities for small-seeded species [32], [33], [41]. However, fire consumption of the organic layer also consumes seeds stored in the seed bank and reduces their potential to establish at a site. In our study area, seed-bank species are relatively uncommon, and we are aware of only one species (*Corydalis sempervirens*) that is an obligate seed-bank species. Nevertheless, this species was positively associated with increases in fire severity, suggesting that the positive effects of improved seedbed quality outweigh negative effects on seedbank consumption even in severely burned sites, at least within organic-rich black spruce forests.

Our results have important biogeographic and functional implications for Alaska’s boreal forest. The most striking differences in community composition occurred between low-severity burns and moderate-to-high-severity burns, mimicking post-fire colonization patterns of tree species [14]. Low-severity fires have predominated in Interior Alaska for several millennia [40]; however there is evidence of a current shift to more high severity fire that is expected to continue in the future [19], [42]. If fire severity continues to increase, our results predict that species that rely on resprouting from surface layers, root in the upper organic layers, or survive in residual patches would likely decrease in abundance on the landscape of Interior Alaska. Affected species include several with an arctic affinity that extend into the boreal forest (e.g., *Betula nana*, *Rubus chamaemorus*, *Oxycoccus microcarpus*, *Eriophorum vaginatum*, and *Rhododendron tomentosum*), as well as many lichens and mosses that currently define and dominate boreal Alaska [25]. In contrast, several species with temperate affinity responded positively to fire severity in our study (e.g. *Populus tremuloides*, *Chamerion angustifolium*, and *Equisetum arvense*) and would likely increase with more severe fires. These responses to increasing fire severity could cause biogeographic shifts to occur more rapidly than would be expected from species’ direct responses to climate. Similarly, the higher productivity of species favored by high severity fire (e.g. those listed above) could amplify the expected stimulation of decomposition and nutrient turnover expected with climate warming [43].

## Supporting Information

Table S1 **Site/Trait Matrix (S×T) derived by multiplying Species/Site matrix (A×S and Species/Trait matrix (A×T) then relativizing data by site so that each set of traits summed to 1.** A**×**T was derived by multiplying the species abundance by its plant trait dominance as measured in the field for each trait. Sites are sorted by fire severity.
(XLSX)

Table S2 **Complete vascular and nonvascular species list across all study sites.** Vascular species were verified in the University of Alaska herbarium and nonvascular species were verified from voucher species in the BNZ LTER working herbarium.
(XLSX)
